# A dataset from a 3-year network of field measurements of soil organic nitrogen mineralization under a mild oceanic temperate climate

**DOI:** 10.1016/j.dib.2021.106795

**Published:** 2021-01-26

**Authors:** Thierry Morvan, Yvon Lambert, P. Germain, Laure Beff

**Affiliations:** a UMR SAS, INRAE, Institut Agro, 35000 Rennes, France; b Chambres d'Agriculture de Bretagne, 35000 Rennes, France

**Keywords:** Soil nitrogen mineralization, Field experimental network, Cropping system, STICS model

## Abstract

Improved assessment and prediction of soil organic nitrogen (SON) mineralization is essential, as it contributes significantly to the nitrogen (N) nutrition of crops and remains a major economic and environmental challenge. SON mineralization is a function of soil properties, land use and climate, which led us to monitor a network of 137 cultivated fields covering the wide diversity of soils, crop rotations and cropping practices throughout Brittany (France). SON mineralization was quantified by the mineral N balance calculated for a maize crop not fertilized with N; it was determined by measuring soil mineral N (SMN) in the 0-90 cm soil profile in March (Ni) and October (Nf) and N uptake by the maize crop, and predicting nitrate leaching (N_leached_) using the STICS model. SMN and plant N uptake were measured in triplicate. To predict N_leached_, STICS was initialized at the date of Ni measurement. In addition, the experimental design was based on estimating SON for three consecutive years (2012-2014) to improve the accuracy of measuring mineralization. An indicator of the cropping system (I_Sys) was developed that integrated well the effects of crop rotation and the frequency of manure application; it can be considered a good index of effects of the cropping system on SON mineralization. This dataset may be used for a variety of applications, such as analysing effects of soil properties, cropping history and climatic conditions on SON mineralization, or evaluating the accuracy of soil-plant models (e.g. STICS, CERES).

**Specifications Table**

**Table:** 

Subject	Agricultural Sciences
Specific subject area	Agronomy and Crop Science
Type of data	Table Figure
How data were acquired	Soil organic nitrogen (SON) mineralization was assessed for three consecutive years (2012-2014) in a network of 137 experimental fields in Brittany (France). The fields were chosen to cover a wide range of soil types under different management systems and climatic conditions. Mineralization of N was quantified by the mineral N balance, which was estimated from March to October for a maize crop not fertilized with N.
Data format	Raw Analysed
Parameters for data collection	Net SON mineralization was calculated from the end of winter to the beginning of the autumn from the mineral N mass balance of an unfertilized maize crop. The N balance was determined by measuring soil mineral nitrogen (SMN) in the 0-90 cm soil profile in March and October and N uptake by the maize crop, and predicting nitrate leaching using the STICS model for three subplots per field.
Description of data collection	SMN content in the soil profile, split into 3 layers (0-30, 30-60 and 60-90 cm), was measured twice a year by composite sampling. Soil inorganic N was extracted by agitation for 30 min in a 1M KCl solution. The NH_4_^+^ and NO_3_^−^ contents of the soil extracts were then determined by continuous flow colorimetry. Aboveground biomass was weighed at harvest to measure crop yields; 5 plants were subsampled per subplot to measure dry matter at 103 °C and N content, which was determined by the Dumas dry-combustion method after drying plants at 50 °C. SMN and plant analyses were performed in triplicate.
Data source location	The data were collected from a network monitored throughout the region of Brittany (France) by the Regional Chamber of Agriculture and INRAE. The dataset provides GPS coordinates of the experimental fields.
Data accessibility	This article provides the analysed data. Raw data are deposited in a public repository. Repository name: Data INRAE Data identification number: https://doi.org/10.15454/VYEYBK Direct URL to data: https://doi.org/10.15454/VYEYBK

**Value of the Data**

• Quantifying SON mineralization and improving knowledge about its main drivers remains an important issue. This dataset provides important benchmarks on soil mineralization in a mild oceanic temperate climate, in a variety of soil and cropping contexts. This dataset was built from a sound experimental design that included three replicates of the measurements for three consecutive years.
• This dataset is of interest to agronomists, soil scientists, statisticians and modellers.
• This dataset can be used in a variety of applications: i) statistical analysis of effects of soil properties and cropping history on the variability in mineralization, ii) analysis of effects of the climate on interannual variability in mineralization, and iii) evaluation of the accuracy of soil-plant models (e.g. STICS, CERES) in predicting yield and N uptake by a maize crop, and dynamics of soil mineral N (SMN).

## 1 Data Description

**Fig. 1:**
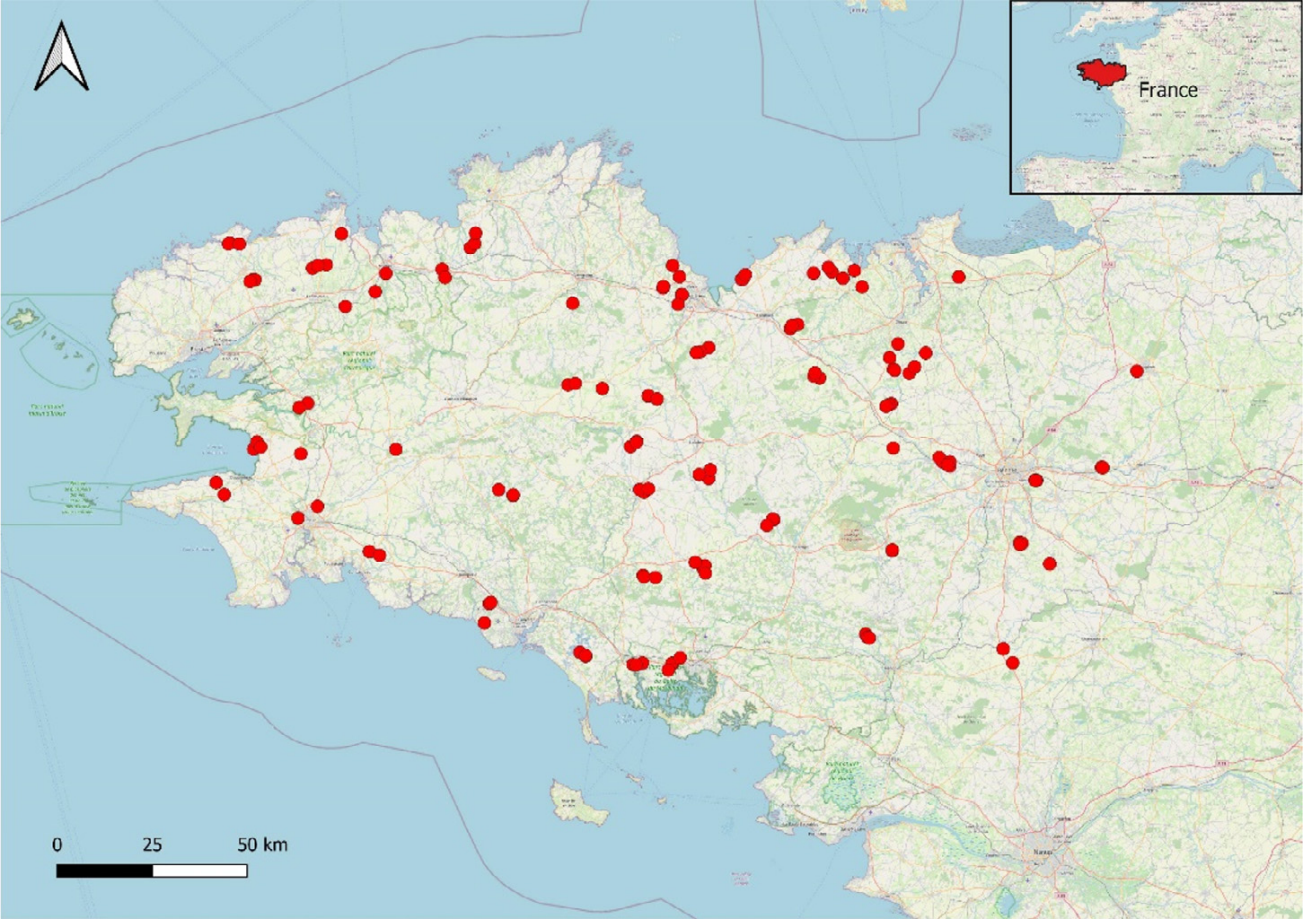
Location of the network fields.

**Fig. 2:**
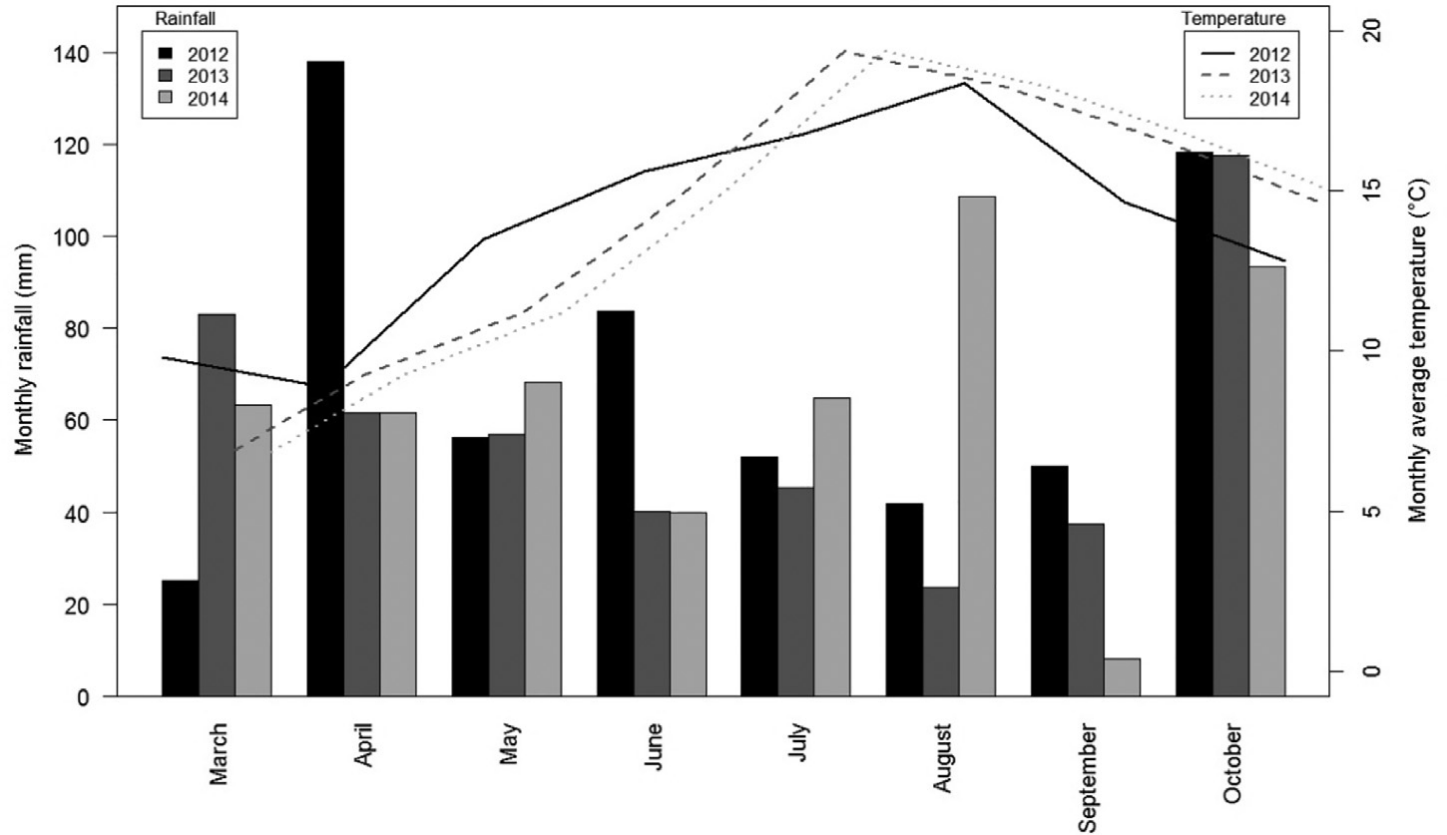
Monthly rainfall and mean air temperature from March-October for the 3 years of the experiment.

**Fig. 3:**
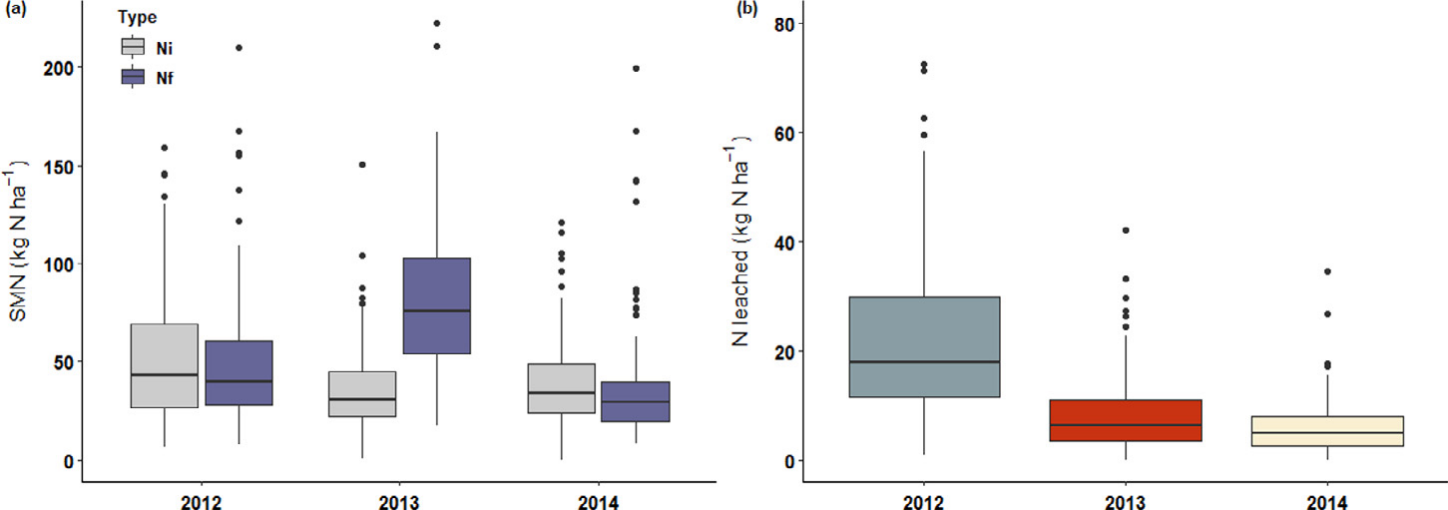
Boxplots of (a) soil mineral nitrogen (SMN) in the 0-90 cm soil profile in March (Ni) and October (Nf) and (b) the estimated amount of N leached from the dates of Ni to Nf for the 3 years of the experiment. (Whiskers extend to 1.5 times the interquartile range).

**Fig. 4:**
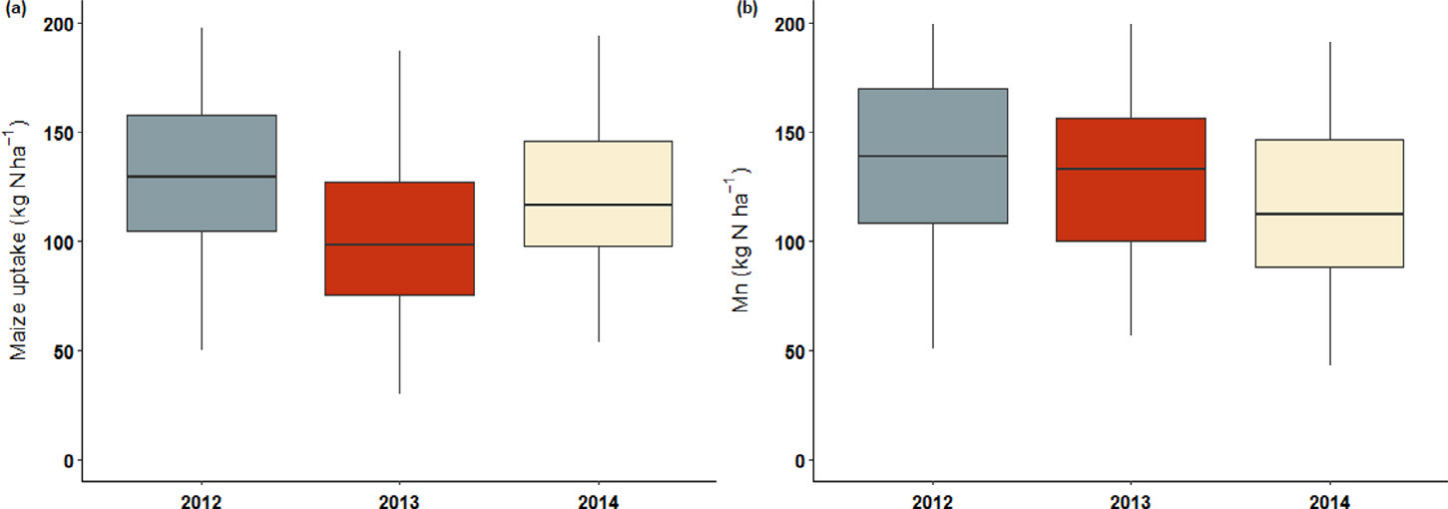
Boxplots of (a) nitrogen uptake by the maize crop and (b) soil organic nitrogen (SON) mineralization calculated from the mineral nitrogen balance from the dates of Ni to Nf for the 3 years of the experiment. (Whiskers extend to 1.5 times the interquartile range).

**Fig. 5:**
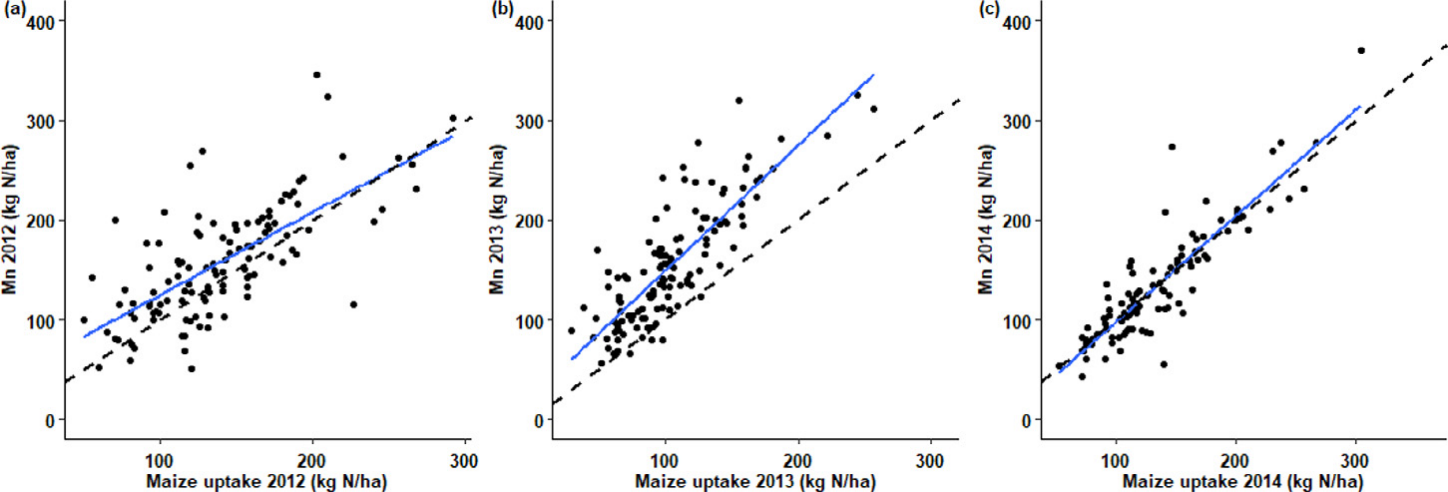
Nitrogen uptake by the maize crop and soil organic nitrogen (SON) mineralization (Mn) for (a) 2012, (b) 2013 and (c) 2014 (the dashed line represents 1:1 line and the solid blue line the regression line).

**Fig. 6:**
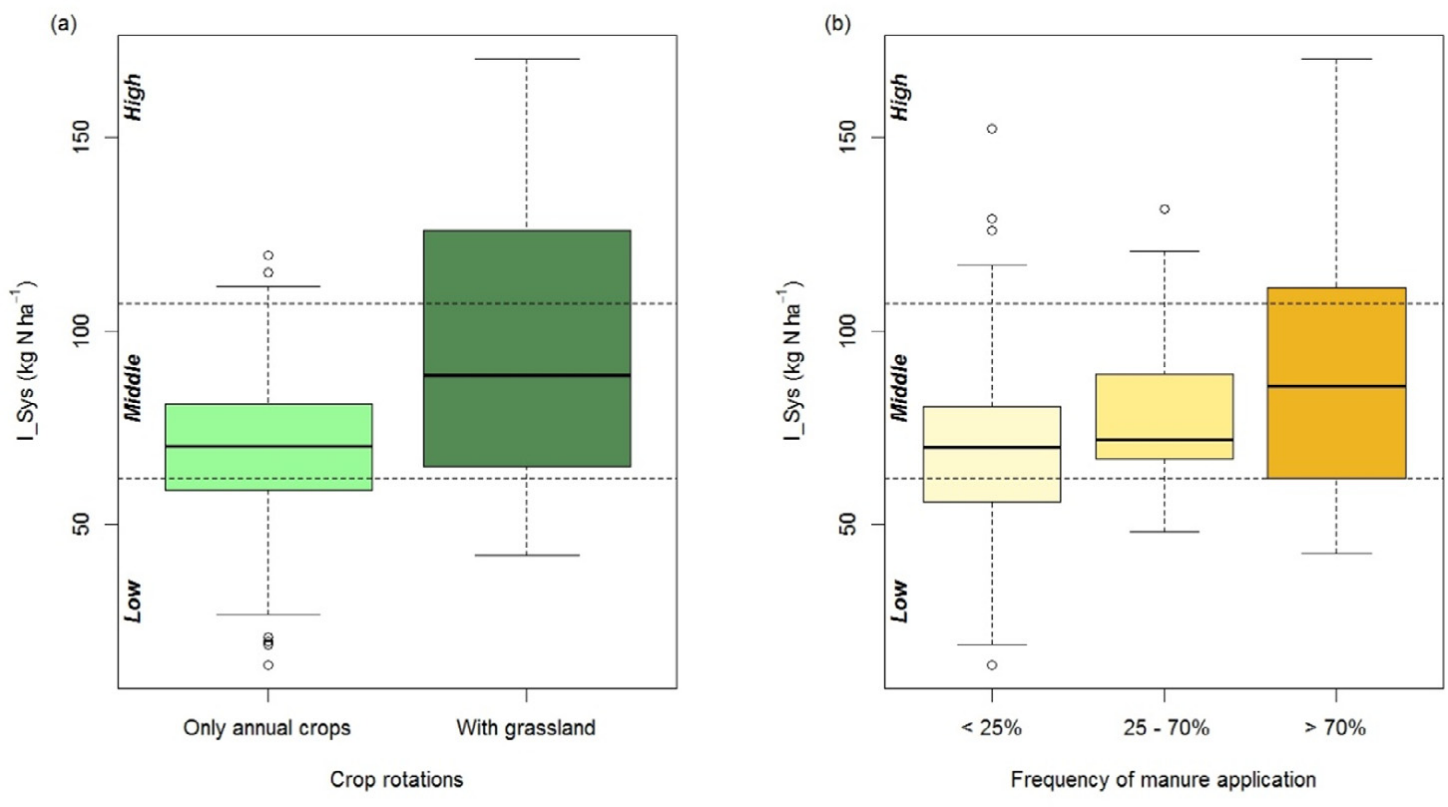
Boxplots of the cropping system indicator (I_Sys) as a function of (a) presence of grassland in the rotation from 1995-2009 and (b) three classes of organic waste application frequency for the 15 years before the beginning of the experiment, ranging from 0% (i.e. no application) to 100% (i.e. application every year). Dashed lines indicate the three levels of I_Sys: Low, Middle and High. (Whiskers extend to 1.5 times the interquartile range).

**Fig. 7:**
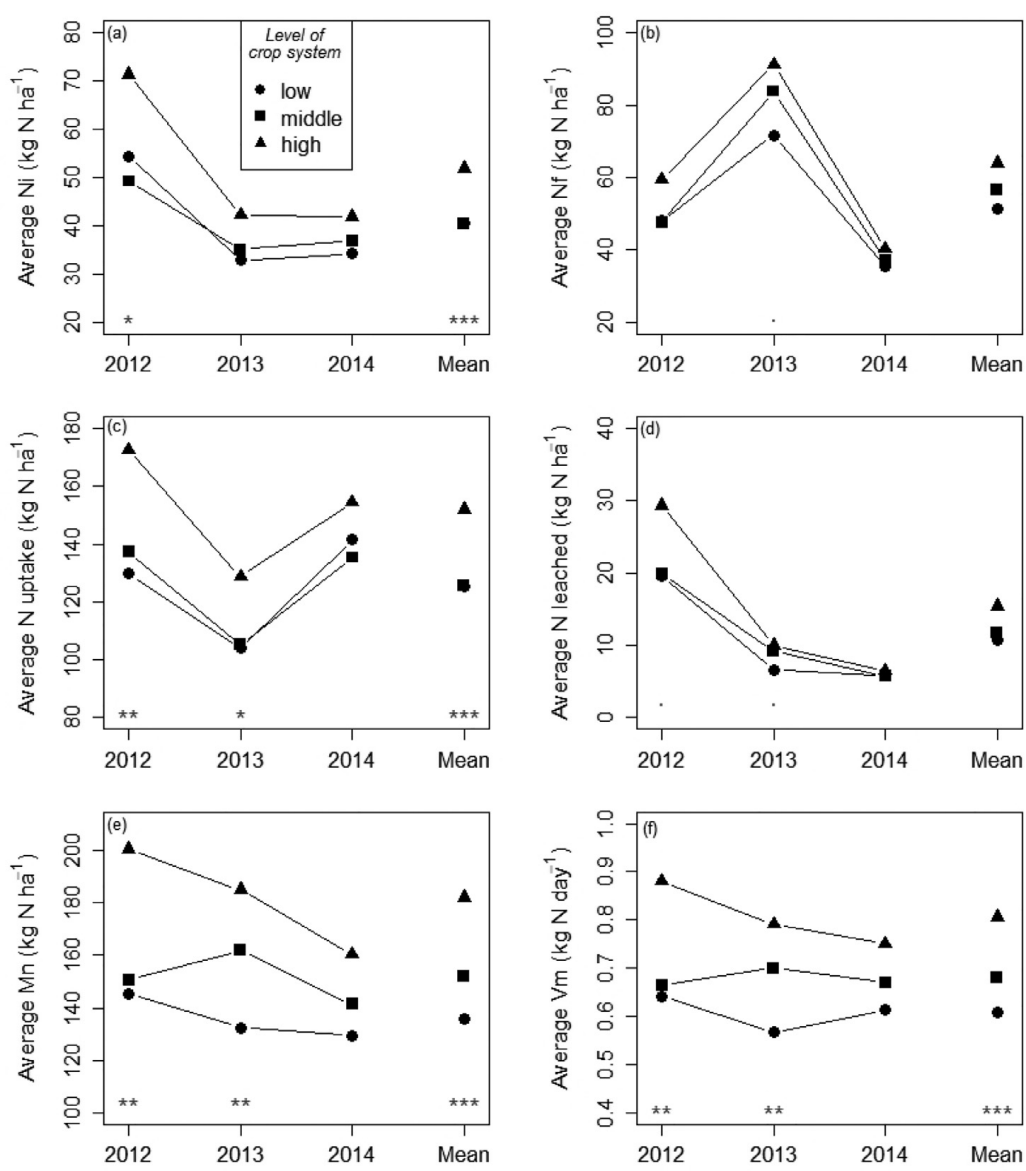
Effect of the level of the cropping system indicator on (a) initial mineral N content, (b) final mineral N content, (c) N uptake by maize plants, (d) N leached, (e), net N mineralization and (f) the daily N mineralization rate (Vm, equal to Mn divided by the number of days from the measurement dates Ni to Nf). The data were averaged for all fields with the same cropping system level each year and for all 3 years. Symbols at the bottom of each graph indicate the significance of the effect of the cropping system level, obtained with the Kruskal-Wallis test: “.” for p < 0.1, “*” for p < 0.05, and “**” for p < 0.01.

This article includes tables and figures that describe data acquired in the network from 2012-2014, including the location of fields (Fig. 1); weather data averaged over the network from March-October for the three years (Table 1, Fig. 2); the mean and variation range of physico-chemical properties of the soils (Table 2); and boxplots of components of the mineral N balance for the three years (SMN and nitrate leaching (Fig. 3), maize N uptake and SON mineralization (Fig. 4)). They also illustrate the large difference in the contribution of maize N uptake to the mineral N balance among the three years (Fig. 5), boxplots of the cropping system indicator developed in this project (Fig. 6) and parameters of the decay-series model used to calculate the indicator of application of organic waste products (Table 3). Finally, they show the significant response of the components of the mineral N balance and of SON mineralization to the level of this indicator (Fig. 7).

**Table 1:** Mean temperature, rainfall and Penman evapotranspiration (PET) from the network fields from March-October for the 3 years of the experiment and for the 1994–2014 period (values in brackets indicate the standard deviation).

Year	Air temperature (°C)	Cumulative rainfall (mm)	Cumulative PET (mm)
2012	13.6	662	534
2013	13.6	527	554
2014	14.3	531	562
1994-2014	13.8 (0.52)	563 (120)	607 (43)

**Table 2:** Physico-chemical properties of the 0-30 cm soil layer of granulometric, N and C contents (g kg soil^−1^), pH and Metson cation exchange capacity (CEC, meq 100 g soil^−1^). SD = standard deviation.

	Contents								
Statistic	Clay	Fine silt	Coarse silt	Fine sand	Coarse sand	N	C	pH	CEC
Mean	194	248	268	126	164	1.81	19.8	6.1	9.93
Min.	124	98	66	4	7	0.87	8.88	4.8	5.5
Max.	408	512	534	273	517	3.86	45.6	7.9	17.2
SD	51.8	87.5	118.8	53.6	129.3	0.57	6.5	0.5	2.53

**Table 3:** Decay-series model coefficients to calculate the I_owp indicator.

Year	Cattle manure	Poultry manure	Pig and cattle slurry
1	0.268	0.370	0.450
2	0.147	0.091	0.249
3	0.081	0.067	0.137
4	0.044	0.050	0.076
…			
n	0.044	0.050	0.076

The dataset is composed of five Excel files that contain raw data (Table 4). It includes data on field location, soil properties, maize yields and N content, SMN values, mineral N balance components, SON mineralization and monthly weather data from March-October for each field of the network*.* The dataset is available via the Data INRAE portal.

**Table 4:** Contents of the dataset.

File name	Variable name	Content
ID_coord_soil_ISys.xlsx	X_WGS84	X WGS84 coordinate
	Y_WGS84	Y WGS84 coordinate
	ID_field	Field identifier
	Clay	Clay content (g kg^−1^)
	Fine_Silt	Fine silt content (g kg^−1^)
	Coarse_Silt	Coarse silt content (g kg^−1^)
	Fine_Sand	Fine sand content (g kg^−1^)
	Coarse_Sand	Coarse sand content (g kg^−1^)
	pH	Water pH
	CEC_Metson	Metson cation exchange capacity (meq 100 g^−1^ soil)
	N	Soil organic N content (g kg^−1^)
	C	Soil organic C content (g kg^−1^)
	I_Sys	Cropping system indicator (kg N ha^−1^)
Maize_crop.xlsx	Date	Date of harvest
	DM	Dry matter (in %)
	Crop_yield	Crop yield (t DM ha^−1^)
	N	Plant N content (g N kg^−1^ DM)
SMN.xlsx	Soil_moisture_0_30	Moisture content of the 0-30 cm soil layer (in % dry soil)
	Soil_moisture_30_60	Moisture content of the 30-60 cm soil layer (in % dry soil)
	Soil_moisture_60_90	Moisture content of the 60-90 cm soil layer (in % dry soil)
	NO3_0_30	Amount of nitrate-N in the 0-30 cm soil layer (kg N ha^−1^)
	NO3_30_60	Amount of nitrate-N in the 30-60 cm soil layer (kg N ha^−1^)
	NO3_60_90	Amount of nitrate-N in the 60-90 cm soil layer (kg N ha^−1^)
	NH4_0_30	Amount of ammoniacal-N in the 0-30 cm soil layer (kg N ha^−1^)
	NH4_30_60	Amount of ammoniacal-N in the 30-60 cm soil layer (kg N ha^−1^)
	NH4_60_90	Amount of ammoniacal-N in the 60-90 cm soil layer (kg N ha^−1^)
Nitrogen_balance_sheet.xls	Date_Ni	Measurement date of Ni
	Date_Nf	Measurement date of Nf
	Ni	Amount of SMN of the 0-90 cm soil profile at date_Ni (kg N ha^−1^)
	N_uptake	Maize N uptake (kg N ha^−1^)
	Nf	Amount of SMN of the 0-90 cm soil profile at date_Nf (kg N ha^−1^)
	N_leached	Estimated N leaching from Ni to Nf dates (kg N ha^−1^)
	Mn	Soil organic N mineralization (kg N ha^−1^)
	Vm	Daily rate of mineralization (kg N ha^−1^ d^−1^)
Meteorological_data.xls	[Rain_March; Rain_October]	Monthly rainfall from March to October (mm)
	[T_March; T_October]	Mean monthly air temperature from March to October (°C)
	[PET_March; PET_October]	Monthly Penman evapotranspiration from March to October (mm)

## 2 Experimental Design

### 2.1 Materials and methods

#### 2.1.1 Network presentation

Experiments were performed for three years in a network of 137 cultivated fields located throughout Brittany, France (Fig. 1). The soil was sampled with an auger in each field to determine its depth, layers and their textural class. In the upper layer (0-30 cm), most soils had a silty loam (n=81) or loamy (n=33) texture. The other soils were sandy loam (n=15), clay loam (n=4), silty clay loam (n=3) and silty clay (n=1). The ranges of variation of the main physico-chemical properties of the soils are presented in Table 2.

Before the experiments, 82 fields had annual crop rotations, 30 fields had grassland in their rotations and the remaining 25 fields were summer fallow or cultivated with vegetables. The main crop rotation was maize-wheat. All annual crop rotations had silage (n=60) or grain maize (n=22). The type, duration and management of the 30 grassland rotations varied among fields, with cutting (n=9), grazing (n=15) or a combination of both (n=6). The duration of grassland in the rotation was usually longer than 5 years (n=15) or approximately 5 years (n=9). Animal waste was regularly applied to half (n=65) of the fields, especially on maize crops; of these fields, 26 received manure every year, with one or two applications per year. Fifty-seven fields received at least one application every 4 years of cattle manure (n=36), pig slurry (n=14), cattle slurry (n=8) or poultry manure (n=5).

The climate in Brittany (France) is mild oceanic temperate, with a pronounced east-west rainfall gradient: mean annual rainfall over the 1994-2014 period ranged from 732 mm in the east to 1376 mm in the west. Mean annual temperature was 11.7 °C, with a monthly mean maximum in August (19.2 °C in the east and 16.3 °C in the west) and minimum in January (4.8 °C in the east and 7.8 °C in the west).

The weather of the three experimental years differed: 2013 was dry, especially in summer, with rainfall that was much lower than the mean monthly rainfall observed from 1994-2014 (Table 1, Fig. 2). In contrast, 2014 was rainy, especially in winter (January and February) and in summer (July and August). The year 2012 was variable, with dry periods in winter (January and February), rainy periods in April and June and rainfall close to the mean in July. In addition to this inter-annual variability, weather varied greatly among fields within a given year.

#### 2.1.2 Experimental design

The objective of the experiment was to quantify N mineralization of the “humified” soil organic matter (SOM), as the N mass balance used in this experimental approach included N mineralization not only from this pool, but also from other pools, including animal waste and annual crop or grassland residues recently incorporated into the soil. To this end, i) the experimental zone was cropped with silage maize for four (since 2011) or five (since 2010) consecutive years without any mineral or organic fertilization and ii) only mineralization data for 2012, 2013 and 2014 were considered, to limit biases resulting from inputs of fertilizers and crop residues incorporated into the soil the year before the start of the experiment. The experimental design was thus based on estimating SON for three consecutive years. Experimental monitoring was performed on an area of 1485 m² (33 m × 45 m), divided into three subplots of 45 m² (6.0 m × 7.5 m) in the middle for replicate measurements.

### 2.2 Calculating net soil N mineralization

Net soil N mineralization (Mn) was calculated from the end of winter to the beginning of autumn from the mineral N mass balance of a maize crop not fertilized with N, as follows:

(1) $$\text{Mn}=\text{Nf}--\text{Ni}+N_{\text{uptake}}+N_{\text{leached}}$$

with Ni and Nf corresponding to the SMN in the 0-90 cm soil profile in March and October, respectively, N _uptake_ corresponding to N uptake by the plant (kg N ha^−1^), and N_leached_ corresponding to nitrate leaching that may occur in spring, after measurement of Ni (kg N ha^−1^). Ni, Nf and N_uptake_ were measured in triplicate.

N_leached_ was estimated using the STICS model [1], which was parameterized with the soil properties of each field, and initialized at the measurement date of Ni. STICS was initialized with the mean soil moisture and mineral N contents of the three replicates, for each soil layer. The field-capacity and wilting-point moisture contents required by the STICS leaching subroutine were estimated from moisture content measured in the field at date Ni (using the method of [2]) and from pedotransfer functions developed by [3], respectively.

Eq. (1) is a simplified approach for estimating the mineral N mass balance, but it is valid in a situation without N fertilization. Gaseous N losses can be assumed to be very low and compensated by atmospheric deposition and symbiotic fixation of N.

### 2.3 Soil and plant analysis

Initial SMN content (Ni) was measured at the end of winter (March), and final SMN (Nf) was measured at the beginning of autumn (October), before resumption of nitrate leaching. Ten soil cores were taken in each subplot, split into three layers (0-30, 30-60 and 60-90 cm), and soil samples for each layer were obtained by mixing its 10 cores to obtain three replicates per layer. The soil samples were transported in a cool box to the analysis laboratory on the day of sampling to extract N from the fresh sample in a KCl solution within 24 hours after sampling. Soil inorganic N was extracted by agitation for 30 min in a 1M KCl solution, and the NH_4_^+^ and NO_3_^−^ contents of the soil extracts were then determined by continuous flow colorimetry by the methods developed by [4] and [5], respectively.

Bulk density was measured once, in triplicate, in 2011, for each experimental field and each of the three layers (0-30, 30-60 and 60-90 cm), using an 8 cm diameter root auger, which cored undisturbed samples of known volume. All samples were oven dried at 105 °C, weighed, and sieved at 2 mm. Samples were then weighed again to determine gravel content. The bulk density of the fine-earth fraction calculated from the dry mass and the core volume was used to convert the mineral N content of the samples to kg N ha^−1^.

The soil of the upper layer (0-30 cm) was sampled in March 2013 to estimate certain soil properties. Each soil sample was obtained by mixing the 10 soil samples from each soil layer from the three subplots, to obtain one composite per field. The soil samples were dried at 40 °C and sieved at 2 mm for physical and chemical analysis. Total C and N were determined by the Dumas dry-combustion method. Cation exchange capacity (CEC) was established using the Metson method [6], and pH was obtained in water [7]. Soil texture was based on measuring the particle size of five fractions: clay (< 2 µm), fine silt (2-20 µm), coarse silt (20-50 µm), fine sand (50–200 µm) and coarse sand (200-2000 µm) [8]. Soil properties of the 137 fields are summarized in Table 2.

Aboveground biomass and N content of the maize crop were quantified at harvest. For all subplots, maize plants were harvested and weighed. Then, five representative plants were subsampled and ground in the field to make a composite that was sent to the lab for dry matter at 103 °C and N content analysis [9]. N measured in aboveground biomass corresponds to the N exported by the plant. Total N uptake of maize was obtained by multiplying N in the aboveground biomass by 1.15 to take into account the amount of N in the roots at harvest [10,11].

### 2.4 Calculating an integrated indicator of the cropping system

An indicator of the cropping system (I_Sys) was calculated to integrate the diversity of field management (crop rotation and organic waste application) among fields, considering a period of 15 years before the year of interest. I_Sys was calculated by summing an indicator (I_crops) of the effect of the N returned to the soil in crop residues and an indicator (I_owp) of the effect of repeated applications of organic waste products on soil mineralization.

I_crops was calculated as the mean N returned to the soil by crops over the 15 previous years. For a given year, the N returned to the soil was estimated as the difference between the N absorbed and exported by the crop. These amounts were calculated using the yield obtained in the field multiplied by the amounts of N absorbed and exported per unit of yield, respectively, provided by national reference standards.

I_owp estimated the additional rate of net N mineralization due to organic waste application as a function of its type, quantity and application frequency using the concept of a decay series, developed by Pratt et al [12]. The decay series is represented by a series of numbers in which the first number represents the percentage of total manure N applied that is available in mineral form during the first year; the second number represents the percentage of residual N from the first year that mineralizes during the second year, and so on [13]. The decay series used for the types of organic waste applied to the network fields before the experiment were obtained by calibrating the model with a French network of medium-term trials (unpublished data) (Table 3). To minimize uncertainty in the parameters, organic N storage and supplemental N mineralization, calculated as the difference from control treatments, were calibrated at the same time [14]. This approach allowed us to modify the concept of the decay series slightly and improve it by replacing the total N availability coefficient for year 1 of the Pratt model with the the mineralization rate of organic N applied. To provide an indication of magnitude of I_owp, biannual applications of cattle manure at a rate of 35 t ha^−1^, biannual applications of poultry manure at a rate of 8 t ha^−1^ and annual applications of pig slurry at a rate of 30 m^3^ ha^−1^ result in I_owp values equal to 22, 17 and 8 kg N ha^−1^, respectively.

I_Sys values ranged from 14-185 kg N ha^−1^ and integrated effects of crop rotation well, especially the presence of grassland in the rotation (p < 0.001, Fig. 6a) and the frequency of organic waste application (p < 0.001, Fig. 6b). We therefore consider it a good index of the cropping system. To assess impacts of I_Sys on N mass balance and soil N mineralization, we classified its values into three levels using k-means clustering: low (≤ 63 kg N ha^−1^), moderate (63-98 kg N ha^−1^) and high (> 98 kg N ha^−1^). These three classes contained 32%, 53% and 15% of the fields, respectively.

## Ethics Statement

Not applicable.

## CRediT Author Statement

**Thierry Morvan:** Conceptualization, Methodology, Validation, Formal analysis, Investigation, Data curation, Writing - original draft, Writing - review & editing, Visualisation, Supervision, Project administration, Funding acquisition; **Yvon Lambert:** Methodology, Validation, Formal analysis, Investigation, Data curation, Supervision, Project administration, Funding acquisition; **P. Germain:** Investigation, Data curation; **Laure Beff:** Validation, Formal analysis, Investigation, Data curation, Writing - original draft, Visualisation.

## Declaration of Competing Interest

The authors declare that they have no known competing financial interests or personal relationships which have, or could be perceived to have, influenced the work reported in this article.
